# Cricotracheostomy for patients with severe COVID-19: A case control study

**DOI:** 10.3389/fsurg.2023.1082699

**Published:** 2023-01-17

**Authors:** Naoki Mukai, Masahiro Okada, Saki Konishi, Mitsuo Okita, Siro Ogawa, Kosuke Nishikawa, Suguru Annen, Muneaki Ohshita, Hironori Matsumoto, Satoru Murata, Yutaka Harima, Satoshi Kikuchi, Shiori Aibara, Hirofumi Sei, Kunihide Aoishi, Rie Asayama, Eriko Sato, Taro Takagi, Kaori Tanaka-Nishikubo, Masato Teraoka, Naohito Hato, Jun Takeba, Norio Sato

**Affiliations:** ^1^ Department of Emergency and Critical Care Medicine, Ehime University Graduate School of Medicine, Toon, Japan; ^2^ Department of Otolaryngology, Head and Neck Surgery, Ehime University Graduate School of Medicine, Toon, Japan; ^3^ Department of Bone and Joint Surgery, Ehime University School of Medicine, Toon, Japan

**Keywords:** COVID-19, SARS-CoV-2, tracheostomy, cricoid cartilage, decannulation

## Abstract

**Background:**

Tracheostomy is an important procedure for the treatment of severe coronavirus disease-2019 (COVID-19). Older age and obesity have been reported to be associated with the risk of severe COVID-19 and prolonged intubation, and anticoagulants are often administered in patients with severe COVID-19; these factors are also related to a higher risk of tracheostomy. Cricotracheostomy, a modified procedure for opening the airway through intentional partial cricoid cartilage resection, was recently reported to be useful in cases with low-lying larynx, obesity, stiff neck, and bleeding tendency. Here, we investigated the usefulness and safety of cricotracheostomy for severe COVID-19 patients.

**Materials and methods:**

Fifteen patients with severe COVID-19 who underwent cricotracheostomy between January 2021 and April 2022 with a follow-up period of ≥ 14 days were included in this study. Forty patients with respiratory failure not related to COVID-19 who underwent traditional tracheostomy between January 2015 and April 2022 comprised the control group. Data were collected from medical records and comprised age, sex, body mass index, interval from intubation to tracheostomy, use of anticoagulants, complications of tracheostomy, and decannulation.

**Results:**

Age, sex, and days from intubation to tracheostomy were not significantly different between the COVID-19/cricotracheostomy and control/traditional tracheostomy groups. Body mass index was significantly higher in the COVID-19 group than that in the control group (*P* = 0.02). The rate of use of anticoagulants was significantly higher in the COVID-19 group compared with the control group (*P* < 0.01). Peri-operative bleeding, subcutaneous emphysema, and stomal infection rates were not different between the groups, while stomal granulation was significantly less in the COVID-19 group (*P* = 0.04).

**Conclusions:**

These results suggest that cricotracheostomy is a safe procedure in patients with severe COVID-19.

## Introduction

Tracheostomy is commonly performed in critically ill patients who require prolonged mechanical ventilation for acute respiratory failure and airway issues. Critical patients with COVID-19 require intensive care unit admission and mechanical ventilation. It has been reported that 8%–16.7% of severe COVID-19 patients require tracheostomy (1–3). Older age and obesity are risk factors for severe COVID-19 (4–9) as well as for prolonged intubation (10); therefore, older and obese patients are more likely to require tracheostomy. However, such patients also tend to have a low-lying larynx and a short neck, which make tracheostomy more difficult. Additionally, COVID-19 is associated with coagulopathy, thrombosis, and bleeding tendency (11), each of which is associated with the potential risk of post-tracheostomy complications.

Cricotracheostomy, a modified procedure for opening the airway through intentional partial cricoid cartilage resection, was recently reported to be useful in cases with a low-lying larynx, obesity, stiff neck, and bleeding tendency (12, 13). As each of these factors is associated with severe COVID-19, we investigated the utility and safety of cricotracheostomy for patients with severe COVID-19.

## Materials and methods

This retrospective study was approved by the institutional review board of Ehime University Medical Hospital (No. 2108029). The need to obtain informed consent was waived because of the retrospective design. Instead, we presented the opportunity to decline to participate in this study to the patients, on our website. This study followed in accordance with the ethical standards of the institutional review board of Ehime University Medical Hospital and with the Declaration of Helsinki.

Patients who were diagnosed with COVID-19 and who required mechanical ventilation and cricotracheostomy at Ehime University Hospital between January 2021 and April 2022 were included in this study. Forty patients who underwent traditional tracheostomy between January 2015 and April 2022 owing to respiratory failure and prolonged intubation not related to COVID-19 were also included in this study as the control group. Patients whose follow-up period was < 14 days were excluded from this study. Data were collected from medical records and comprised age, sex, body mass index (BMI), use of anticoagulants peri-operatively, interval from intubation to cricotracheostomy/tracheostomy, mortality, decannulation, and complications of tracheostomy.

**Figure 1:**
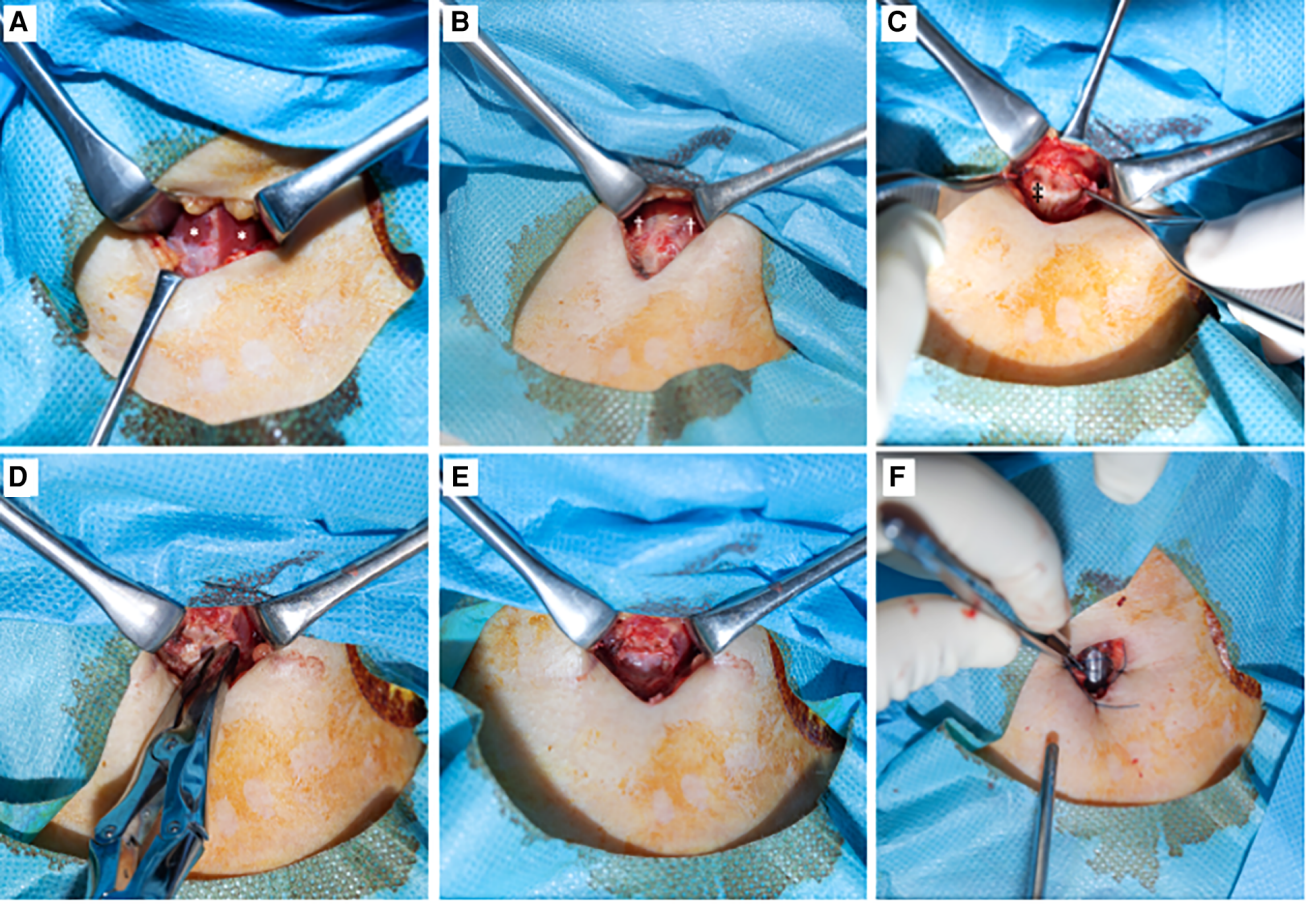
Surgical procedure of cricotracheostomy. (**A**): After vertical skin incision. Bilateral muscles sternohyoideus were exposed. *: muscles sternohyoideus. (**B**): Muscles sternohyoideus were divided at midline. Muscles cricothyroideus were observed. †: muscles cricothyroideus. (**C**): Cricoid cartilage was observed after dissected perichondrium. ‡: cricoid cartilage. (**D**): The anterior portion of the cricoid cartilage was removed. (**E**): After cricoid cartilage was removed, tracheal mucosa was observed. (**F**): An incision was made in the membranous tissue and the flap was sutured to the skin. Endotracheal tube was observed in the trachea.

Among patients with COVID-19, each tracheostomy operation was performed under general anesthesia at bedside in the ICU. A vertical skin incision was made in the neck, and the cricoid cartilage was exposed. The anterior portion of the cricoid cartilage was removed. An incision was then made in the membranous tissue, and the flap was sutured to the skin. After fenestration of the trachea, an endotracheal cannula was inserted. Dividing or retracting the thyroid isthmus was not required in all cases (Figure 1). All cricotracheostomies were performed in negative-pressure ventilation rooms. In the control group, traditional tracheostomy was performed by otolaryngologists under general anesthesia in the operative room. In both groups, anticoagulants were discontinued 6 h prior to surgery and re-started 24 h after surgery.

All data are expressed as the mean and 95% confidence intervals (CIs). Each parameter was compared between the two groups using the two-tailed *t*-test or Fisher's exact test with JMP software for Macintosh (SAS Institute Inc., Cary, NC). A *P* value of < 0.05 was considered statistically significant.

## Results

Twenty patients with severe COVID-19 underwent cricotracheostomy from January 2021 to April 2022. Of these 20 patients, 5 were excluded from this study because the follow-up period was < 14 days (3 died and 2 transferred to another hospital). The remaining 15 patients with COVID-19 who underwent cricotracheostomy were included in this study. Additionally, 40 patients who underwent traditional tracheostomy were included as a control group.

**Figure 2:**
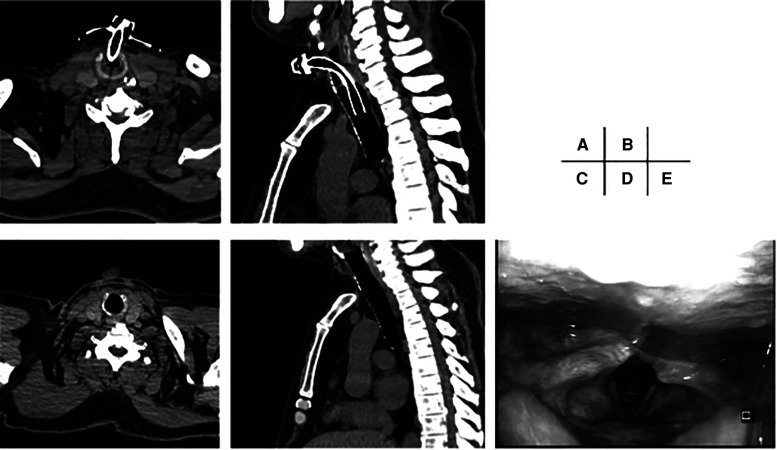
Ct scans and endoscopic image of larynx before and after decannulation. (**A,B**): CT scan before decannulation. The cannula is inserted at the level of the cricoid cartilage. (**C,D**): CT scan three months after decannulation. No stenosis is observed. (**E**): Endoscopic image of larynx three months after decannulation. No laryngeal stenosis is observed.

The patients' clinical characteristics are summarized in Table 1. The mean age was 69 years (95% CI, 61.2–72.0) in the COVID-19 group and 69 years (95% CI, 62.3–70.2) in the control group; there was no significant difference between the groups (*P* = 0.92). Of the 15 patients in the COVID-19 group, 3 (20.0%) were female; 13 (32.5%) were female in the control group. The mean BMI was 25.6 kg/m^2^ (95% CI, 22.8–28.3) in the COVID-19 group and 20.5 kg/m^2^ (95% CI, 19.6–23.2) in the control group. The mean BMI in the COVID-19 group was significantly higher compared with the control group (*P* = 0.02). All patients in the COVID-19 group received anticoagulants, while 9 (22.5%) received anticoagulants peri-operatively in the control group. Anticoagulants were used significantly more often in the COVID-19 group compared with the control group (*P* < 0.001). Prone therapy after tracheostomy was initiated in 10 (66.7%) patients in the COVID-19 group, while no patients received prone therapy in the control group (*P* < 0.001). Days from intubation to tracheostomy, follow-up period, and mortality rates were not significantly different between the groups (*P* = 0.71, > 0.99, and 0.34, respectively). Decannulation was achieved in 12 (80.0%) patients in the COVID-19 group and in 13 (32.5%) patients in the control group. The decannulation rate was significantly higher in the COVID-19 group compared with the control group (*P* = 0.002). Eight (66.7%) of the 12 patients in the COVID-19 group who achieved decannulation did not require a surgical procedure for closure of the stoma, and 7 (53.9%) of the 13 patients in the control group who achieved decannulation did not require a surgical procedure for closure of the stoma. The spontaneous closure rate was not significantly different between the groups (*P* = 0.69). The remaining patients who achieved decannulation underwent surgery to close the stomas. Among patients who achieved decannulation, no laryngeal or tracheal stenosis occurred in either group (Figure 2).

**Table 1:** Clinical characteristics of the patients.

	COVID-19 (*N *= 15)	Control (*N *= 40)	*P*-value
Surgical procedure	Cricotracheostomy	Conventional tracheostomy	
Age (years)	69 (61.2–2.0)	69 (62.3–70.2)	.92
Sex (male/female)	12/3	27/13	.51
BMI	25.6 (22.8–28.3)	20.5 (19.6–23.2)	.02
Use of anticoagulants, *n* (%)	15 (100%)	9 (22.5%)	<.001
Prone therapy after tracheostomy, *n* (%)	10 (66.7%)	0 (0%)	<.001
Days from intubation to tracheostomy (days)	13 (11.9–13.8)	11.5 (10.7–14.0)	.71
Follow-up period (days)	260.9 (179.2–342.7)	102.1 (55.5–148.7)	.001
Mortality, *n* (%)	3 (20.0%)	14 (35.0%)	.34
Decannulation, *n* (%)	12 (80.0%)	13 (32.5%)	.002
Spontaneous closure of the stoma, *n* (%)	8/12 (66.7%)	7/13 (53.9%)	.69
Comorbidities Bleeding, *n* (%)	2 (13.3%)	4 (10.0%)	.66
Stomal granulation, *n* (%)	1 (6.7%)	15 (37.5%)	.04
Subcutaneous emphysema, *n* (%)	0 (0%)	1 (2.5%)	>.99
Infection, *n* (%)	0 (0%)	2 (5.0%)	>.99

Values are means (95% CI).

BMI, body mass index; CI, confidence interval.

Bleeding from the stoma after surgery was observed in 2 (13.3%) patients in the COVID-19/cricotracheostomy group. These two patients underwent cricotracheostomy during the use of extracorporeal membrane oxygenation. Four (10.0%) patients in the control/traditional tracheostomy group developed post-operative bleeding from the stoma. There was no significant difference in this complication between the groups (*P* = 0.66). The bleeding was not severe, and no surgical procedure or blood transfusions were required in all six patients. Stomal granulation was seen in only 1 (6.7%) patient during the follow-up period in the COVID-19 group, while this was seen in 15 (37.5%) of the patients in the control group. Stomal granulation was significantly more common in the control/traditional tracheostomy group compared with the COVID-19/cricotracheostomy group (*P* = 0.04). Subcutaneous emphysema and infection were rare, and the rates of these complications were not significantly different between the COVID-19 and control groups (*P* > 0.99 and > 0.99, respectively). No pressure injuries involving the skin were not observed in either of the groups.

## Discussion

Tracheostomy in COVID-19 patients is reported to have several positive effects: 1) Tracheostomy decreases the risk of mortality (14, 15), 2) shortens the intensive care unit stay (14–16), 3) decreases the use of sedative drugs (17), and 4) improves mental status as measured by the Richmond Agitation-Sedation Scale score (18). Additionally, the rate of infection transmission to healthcare workers during tracheostomy is extremely low when appropriate personal protective equipment is used (19). It has also been reported that tracheostomy at appropriate timing should be considered to avoid laryngotracheal stenosis (20–24).

Older age and obesity are risk factors for severe COVID-19 and prolonged intubation (4–10). These risk factors are also related to a low-lying larynx, short neck, and stiff neck. Additionally, severe COVID-19 patients are more likely to have bleeding tendencies (11). Each of these factors is considered to increase the risk of tracheostomy complications, including bleeding. In this study, BMI was significantly higher and the rate of use of anticoagulants was higher in the COVID-19 group compared with the control group. Therefore, to avoid the risk of bleeding and other complications, we chose cricotracheostomy as an alternative to conventional tracheostomy for all patients with severe COVID-19 who required tracheostomy.

Cricotracheostomy offers several advantages over conventional tracheostomy (12, 13). First, conventional tracheostomy can be difficult to perform at the proper level (i.e., the second or third tracheal ring) in patients with a low-lying larynx or a short neck. In such patients, cricotracheostomy can reduce the likelihood of cricoid distress induced by cannulation, which is a possible cause of subsequent laryngeal granulation and stenosis. In our cohort, the degree of stomal granulation was significantly less in the COVID-19/cricotracheostomy group compared with the control group. Second, the cricoid cartilage is a reliable landmark as it is easily palpated; this makes opening the airway faster and easier compared with conventional tracheostomy, especially in obese patients. Additionally, because the distance from the skin to the trachea is shorter with cricotracheostomy compared with conventional tracheostomy, it is easier to exchange the endotracheal cannula, which may reduce the risk of infection and granulation, or to replace the cannula after accidental removal. In 10 (66.7%) of the COVID-19 patients, prone therapy was initiated after cricotracheostomy. As accidental removal of the cannula can occur easily when patients are turned to the prone position, interventions that make cannula replacement easier are worth considering when prone therapy is administered. Third, whereas conventional tracheostomy usually requires division or retraction of the thyroid isthmus, cricotracheostomy does not require these procedures. As post-operative bleeding, which is a major complication of tracheostomy, occurs often from the inferior thyroid vein (25), cricotracheostomy, which requires less interference in the thyroid area, may reduce the possibility of post-operative stomal bleeding. In many severe COVID-19 patients, clinicians cannot respond to post-operative stomal bleeding immediately because patients are usually isolated, and clinicians must use full personal protective equipment. Additionally, treatment for stomal bleeding may increase the risk of healthcare worker infection. The reported rate of post-operative stomal bleeding in patients with COVID-19 ranges from 2.6% to 30% (26–31). In this study, the bleeding rate was 13.3% (2 patients) in the COVID-19/cricotracheostomy group, which was similar to the rates in previous reports (26–31). However, these two bleeding events were not severe, and no surgical procedure or blood transfusion was required. Additionally, these two patients underwent cricotracheostomy during the use of extracorporeal membrane oxygenation. It is possible that cricotracheostomy reduces the likelihood of peri-operative stomal bleeding in patients with COVID-19.

Performing cricothyrotomy and tracheostomy too high in the trachea is associated with a high incidence of laryngotracheal stenosis (32). It has been reported that iatrogenic etiology, including tracheostomy, is the main cause of laryngotracheal stenosis, and cartilage injury has a potential risk of laryngotracheal stenosis (33, 34). Murono et al. reported that cricotracheostomy has acceptable decannulation outcomes relative to those of conventional tracheostomy (13). Kano et al. reported that the stomas closed without stenosis after cricotracheostomy in three of seven patients (12). In our cohort, 12 (80.0%) of the 15 patients with COVID-19 who underwent cricotracheostomy achieved stoma closure without stenosis; the other three died prior to weaning from ventilation. This result is consistent with the results reported by Murono et al. (13). However, there are few studies regarding the efficacy and the safety of cricotracheostomy. Further studies are needed to clarify this issue. In addition, it has also been reported that care after the tracheostomy procedure influences the outcome of tracheostomy; size of endotracheal tube and cannula, cuff pressure, ischemic condition, length of endotracheal intubation, and prone positioning have been reported to be associated with laryngotracheal stenosis (35, 36). Further studies are needed that consider these factors.

The stomal granulation was significantly lower in the cricotracheostomy/COVID-19 group. The reason for this is unknown, however, it may be related to the shorter distance between skin and trachea in cricotracheostomy. In addition, the administration of systemic corticosteroid may influence the rate of stomal granulation. Systemic corticosteroid is recommended in patients with severe COVID-19 (37), and almost all patients with severe COVID-19 in this cohort were administered systemic corticosteroid. Although it is still unknown whether systemic steroid influences the stomal granulation (38), the lower rate of stomal granulation might be influenced by systemic corticosteroid. In addition, the decannulation rate was significantly lower in the control group. This difference in closure rates may have influenced rates of complications, especially in stomal granulation.

In our cohort, no pressure injuries involving the skin were observed in either of the groups. It has been reported that tracheostomy-related pressure injury is most commonly involves skin in the peristomal area (39). As the stoma site is higher in cricotracheostomy compared to conventional tracheostomy, there is a possibility of a higher predisposition to skin ulcer in cricotracheostomy. As the number of patients in this study was small, further large-scaled studies are needed to confirm whether pressure injuries involving the skin differ between cricotracheostomy and conventional tracheostomy.

This study has several limitations. First, the design was retrospective, and the number of patients was small. A large-scale study is needed to confirm the efficacy and safety of cricotracheostomy for COVID-19 patients. Second, as the observation period was short in this study, more studies with longer observation periods are needed to determine whether stenosis of the larynx is more or less likely with cricotracheostomy. Third, we could not compare the efficacy and safety of cricotracheostomy compared to traditional tracheostomy among patients with severe COVID-19, since we only performed cricotracheostomy in severe COVID-19 patients. In addition, we could not compare patients who underwent cricotracheostomy between COVID-19 patients and a control group, since there were few patients who underwent cricotracheostomy in the control group in this period. Finally, there is a possibility of risk of bias since the inclusion period differed between the COVID-19 group and the control group. As our hospital stopped accepting emergencies other than COVID-19 patients during this pandemic, there were few patients who underwent conventional tracheostomy in the control group during the COVID-19 pandemic. It has been reported that quality improvement in tracheostomy care has improved the quality and safety of tracheostomy (40, 41). Although tracheostomy care in our hospital has not changed over the period of this study, the difference in inclusion period between the COVID-19 group and the control group may influence the outcome of tracheostomy.

## Conclusion

In conclusion, we performed cricotracheostomy in patients with severe COVID-19 and observed minimal complications and no laryngeal stenosis. Cricotracheostomy may be a useful surgical alternative to conventional tracheostomy in patients with severe COVID-19.

## Data Availability

The raw data supporting the conclusions of this article will be made available by the authors, without undue reservation.
